# Practical Points in Practice

**Published:** 1903-12-15

**Authors:** C. C. Noble


### PRACTICAL POINTS IN PRACTICE.

BY C. C. NOBLE, D.D.S.

Read before the Central Michigan Dental Association, November 4, 1903.

In presenting this paper I do not claim that the matter therein contained is essentially new, but I have found the methods set forth of great help to me, and I trust some of the points will be helpful to the members of this Society.

The first subject I will speak of is trichloracetic acid in the treatment of chronic abscess, more especially where we have a fistulous opening. I think we all have had more trouble with this class of cases than with most any other condition we are called upon to treat. My attention was called to this treatment at one of our Detroit Dental Society meetings. I am sorry I can not recall the name of the doctor who spoke of it. After two years' trial I can vouch for the efficacy of the treatment in the great majority of cases. In fistulous openings we have a condition that is impossible to cure unless we get rid of the dead tissue lining the opening, and also destroy the sac at the apex of the root.

Trichloracetic acid is a strong caustic and burns the tissues to a considerable depth, eating away the diseased tissue and sac, giving us a healthy surface that will heal very quickly.

My method of applying it is to take a broach wound with a wisp of cotton, dipped in the acid, and pump the acid up through the opening on the gum. Care should be taken not to allow any of the acid to come in contact with the mucous membrane of the mouth, as it will burn and cause considerable discomfort. We do not get any of this trouble when careful, and the small amount coming through the opening on the gum does not trouble the patient, although it causes a whitening of the tissues immediately surrounding the opening.

After I have injected the acid I seal the tooth and dismiss the patient for two or three days; then remove the stopping and thoroughly wash out with peroxide of hydrogen, and repeat the treatment. Almost all cases will yield to two treatments. I would fill the root and tooth after the second treatment and expect, in a week at least, to find the fistula thoroughly healed.

Removal of the pulp with pressure anesthesia is sometimes rendered very tedious, by reason of the excessive hemorrhage. This can be overcome almost entirely if we will use adrenalin chloride with our cocain. Put a crystal of cocain in the cavity and cover with a very small pledget of cotton soaked in adrenalin chloride and press home with soft rubber. When the pulp is removed it will be found blanched almost white, and sometimes there will be absolutely no blood in the canal. I am indebted to Dr. Worboys for this hint.

In the removal of a pulp that has died and become soft and disintegrated, a wisp of cotton wound on a broach will be found very effectual in removing it. By gently twisting the broach with cotton we entangle the shreds of the pulp in the cotton and can remove the whole pulp at once.

A great deal has been written on root-canal filling, and each one has his favorite method and filling material, but I should like to call to your attention a preparation that I have found, after three years' trial, to be an excellent thing where we cannot remove all of the pulp from the cavity, and I claim this is a condition that the majority of us meet with very often. We read in our journals and hear men say at our meetings that they always get all the pulp out of a tooth. They may be honest in thinking they do, but, just the same, I do not believe they do. It is one thing to demonstrate it on a tooth out of the mouth and an entirely different proposition before extraction. I have tried a great many preparations and have had the best results with a German preparation, Dr. Scheure's Formaldehyde Paste, sold by Gust. Scharmann, New York. After removing all the pulp possible, I force a little of the paste into the canal and then fill with gutta-percha. The paste is recommended as a root-canal filler and the whole canal may be filled with it, but I prefer to rely on gutta-percha for the bulk of the filling.

To those of you who are in the habit of making base plates for taking the bite for artificial dentures, I should like to recommend modeling compound in place of gutta-percha or wax. I know that many dentists use just a roll of wax without any base plate, but I am sure we will all admit that a more accurate bite can be obtained by using a base plate, and it is a difficult operation in many cases, under the most favorable circumstances. Modeling compound makes a very rigid base and is quickly and accurately adapted to the model, being much more easily worked than gutta-percha. It hardens very quickly, and the edges can be easily trimmed and smoothed with a knife, giving us a trial plate that fits the mouth perfectly and does not soften or change shape from pressure.

We are often called upon to repair a rubber plate that is so badly broken that it is a question whether a new plate would not be preferable, the question of expense to the patient, or time, being the only objection. The articulation being perfect, an entirely new denture can be made with very little trouble and almost as quickly as we can repair the old one, by the following method: Cement the pieces together as accurately as possible and insert in the lower half of the flask, keeping the plaster even with the edge of the rim of the plate. Oil the surface of the plaster, rubber and teeth, and build more plaster around the outside of the denture and up even with the occluding edges of the teeth; when this is sufficiently hardened, oil this surface, place the other half of the flask in position, fill with plaster and allow to harden. The flask will separate very easily, leaving the plaster rim around the outside of the teeth, which we next remove and place in the upper part of the flask. By heating the old denture the rubber can be easily removed, leaving the impression of the palatine portion of the mouth perfect. Remove the teeth from the old denture and place in the impressions in the upper part of the flask and proceed to pack the rubber as in any other case. When finished, we have an entirely new denture with comparatively little work.

In bridge-work we often wish to confine the solder to one particular place and not allow it to flow over the adjacent parts. If you will paint the parts you wish to protect with yellow ochre water color, it will prevent the solder flowing over these parts; whiting will also work the same way, but when the heat is applied the whiting flakes off, which is not the case with the yellow ochre.

I have received a great many letters asking me for my experience with the Hurd Twentieth Century Gas Inhaler, and, as a recent inquiry was from a member of this Society, a few words on the subject may be of interest to the rest of you.

I have used the inhaler for over three years, and consider it the best method of administering gas I have ever seen. I have given it a great many times and have never had any bad results from it, although I have had failures, that is, patients that I could not get fully under the influence of the gas; but I do not think we should condemn the apparatus because it will not work every time, as I know of nothing we use, either as a medicine or in our operations, with which we do not sometimes have failures. The hood simply covers the nose, the mouth being propped open and the oral cavity exposed to full view all the time. When the patients are under the influence of the gas, we *keep them under* until our operation is done, and need have no fear of their coming to, as we do with the old method, as we have to remove the hood before we can do any work, and work against time. For the preparation of sensitive cavities, the grinding of a sensitive tooth, or the removal of a pulp it is unsurpassed; in fact, these operations are almost impossible with the old method. I have kept patients under from six to eight minutes, and believe there is no limit to the time. I think the amount of nitrous oxide used is about the same as with the Long apparatus, and also the time required to get a patient fully under; of course this varies, being from half a minute to a minute and a half. The patient inhales a mixture of the nitrous oxide and air, which our text-books say will prevent anesthesia. It certainly will with the Long apparatus, but works well in this way of administration, why, I have as yet been unable to ascertain. I very often, at the beginning of administration, hold the towel over the mouth, but I do not believe it necessary; I do it because I can not get over the feeling that they will go under quicker, although when I have not done so the results are perfectly satisfactory.

As soon as the patients become unconscious, the tongue drops back and compels them to breathe through the nose. We very seldom get the bluish cast to the skin and livid lips that you do with the old method, the patients hardly changing color at all—which is due, I think, to our not getting them into as profound an anesthesia as required with the old method, where it was *necessary* to get a deep anesthesia, as the administration ceased as soon as we began to operate, which is not the case with this apparatus.

DISCUSSION.

Dr. Hathaway: I have had trouble in forcing the mummifying posts into the fine canal, at least I am not sure that I get into the fine and crooked canals. I would like to know how Dr. Noble does this.

Dr. Noble: I mix the paste to a consistency that will flow easily and introduce it into the canal with a suitable probe or broach and then force it with a broach on which cotton is wound so that it makes a piston. A ball of cotton will in some cases be sufficient if placed over the root opening and then pressed with a suitable blunt instrument, such as a burnisher.

Dr. Hines: I have found that the tendency of porcelain facings to discolor when the backings are soldered to place may be prevented by painting the surface of the facing upon which the backing is placed with a thin solution of whiting in water, being careful, of course, not to use enough to interfere with a close adaptation of the backing.

Dr. Worboys: I have experienced some trouble in making seamless crowns, because in drawing down the thimbles the crowns would be so thin that they would not wear long. I overcome this now by using No. 28 plate to start with, and using a size smaller punch than is usually employed until the last hole is reached. When the thimble is put through this hole twice, the first time with a punch one size too small and then follow with the punch fitted for this hole and No. 30 gauge plate. By this means the top of the cap is not thinned at all by stretching.

The discoloration of facings during soldering will usually not take place if the backing fits perfectly and the work is made clean before investment for soldering. I take a great deal of satisfaction in backing facings with gold and platinum plate, that is plate made by sweating gold onto platinum; it is gold on one side and platinum on the other. If I want a yellow effect in the facing I put the gold next the porcelain, if I wish a blue effect place the platinum next the facing. This backing can be used quite thin, and is readily adapted and will not burn off as easily as a gold backing. I think it a great advantage in bridge and some crown-work.

I think most dentists do not appreciate the strain put upon bridges in the molar and bicuspid regions of the mouth. A bridge made by uniting the edges of the backings with solder will not, as a rule, be sufficient to support the strain to which it will be subjected. It will either bend and draw the teeth out of occlusion and cause irritation or break and go to pieces. Wherever it is necessary and possible

I place a stiff bar from one abutment to the other and solder the dummies securely to it.

DR. HOFF: I was particularly interested in the method of repairing vulcanite plates as suggested by the essayist. This is a part of our work that it seems to me we do not give the attention it deserves. We repair any kind of an old rubber plate, it matters not how old or badly-fitting it may be, with little or no thought as to whether it is the best thing to do for our patients. A rubber plate that has been worn ten years ought not as a rule to be repaired, and one that has been vulcanized four or five times is not fit to put into a patient's mouth. The method here suggested of taking out all the old rubber and replacing it with new rubber appeals to me as a very proper and hygienic proceeding. I would go a step farther than the essayist and take a new impression and refit the plate. In this way you give the patient the best possible repair, and it can be done with little more trouble and expense than to make repairs in the ordinary way. If we had a flask with the middle ring in three sections it would make such a proceeding simple, especially for partial plates or where there were deep undercuts.

